# Miasmatic Modifications of Inflammation of the Lungs

**Published:** 1853-05

**Authors:** A. T. Foster

**Affiliations:** Western Branch, Norfolk Co.


### SELECTIONS.

From the Stethoscope.

*Miasmatic Modifications of Inflammation of the Lungs.* By A. T. Foster, M. D.

This is a miasmatic region. During the latter part of the summer and throughout the autumn, scarcely a family escapes an attack of intermittent or remittent fever, while in many cases whole families are simultaneously affected. At all seasons sporadic instances may be noticed, chiefly occurring in those individuals who have been previously attacked. Relapses are frequent: they have been much more so during the past fall than is usual.

Very many patients are not subjected to any treatment; many are treated without medical advice, simply by the administration of quinine; some by cathartics, (mostly Peters' Pills,) others again, by stimulants of various kinds, principally, however, such as are suggested by Thomsonian views. Still another division are treated according to the best judgment of regular practitioners, but before health is entirely restored, discontinue all sanitary or precautionary measures. And again, there is another class, who, suffering from the same influence, exhibit no completely formed attack of fever, but drag through the fall with engorged viscera, or sub-inflammatory hepatic, splenic, duodenal or stomachic derangements, often presenting an irregular collection of symptoms, constituting an anomalous disease; a proteus in form; an ignis fatuus to the search.

Erysipelas is common, almost invariably attended with a weak, quick pulse, epigastric tenderness, a red, angry looking tongue, deficiency of bile in the alvine evacuations, and obstinately persisting dryness of the skin. Its usual termination is in suppuration. Since the visitation of Asiatic cholera in 1849, there have been several severe epidemic prevalences of diarrhœa and dysentery, occurring in the spring and summer. Perhaps it would be more correct to say that, in the spring and early summer of the last three years, these diseases have prevailed extensively.

From early in May to the last of December, however, there are few fatal cases of sickness, taking into the account all the causes of death. Indeed, the mortality will compare favorably with that of any location with which we have had an opportunity of contrasting it. This may appear to some a startling assertion, but we make it with confidence. It is the time when the seeds of death are sown; the harvest is gathered during the four remaining months.

During these four months inflammatory diseases prevail. Those which most frequently fall under the care of a physician, are capillary bronchitis, pneumonia and pleuritis. Ordinary bronchitis is generally a subject for domestic practice. Pleurisy, when uncomplicated, is generally productive of but little trouble to the physician. Possibly this may in part depend upon the fact that the pain leads the patient to apply at once to a medical man. Be this as it may, it is oftener uncomplicated than pneumonia or grave bronchitis. We mean when the pleurisy is the principal affection.

But, during six years, we have not met, in this neighborhood, with a single case of pneumonia or capillary bronchitis, uncomplicated by sufficient disorder within the abdominal cavity, to demand the most prominent attention. This remark does not apply to cases produced by mechanical injury or arising from tubercular phthisis; although it might, likewise, in a qualified degree, be extended to many of these.

It is rare to meet with a case, which, if seen early, will not be, more or less, immediately relieved by vs. The heat, the restlessness, the dryness of skin, the thirst, the difficulty of breathing, the perverted ratio of the respiration to the pulse, the harshness of cough, all acknowledge its beneficial agency; but the patient will rarely bear a sufficiently large abstraction of blood to render this improvement permanent. Still less frequently can a repetition of vs. be carried to any extent, without inducing fearful prostration, either immediately or in the after period of the illness. Cupping is better borne, but many of our patients suffer much from the necessary exposure of the chest, being confined in uncomfortable apartments. A secondary difficulty is, that cupping in the country requires the time of the physician, a circumstance of some weight when the days are short, the roads and the weather bad, and there is a stress of practice.

The irritability of stomach is often such, from the first, as to forbid the use of tartar emetic in any doses. The appearance of the tongue, of a fiery red, that of the fauces and mouth often studded with points of follicular inflammation, would deter us from its employment. Almost invariably the bowels, irregular in action at the onset, become after a while affected by a distressing diarrhœa.

The prompt action of mercury, more particularly a little later in the disease, when it seems most clearly indicated, is limited by the want of a clear index of its action, from the difficulty of knowing how much of the mouth and throat affection depends upon its employment, and by the dread of censure, there being always enough wise ones around to attribute anything, leaving the least room for doubt in the vulgar mind, to its action, while we know that hemorrhage, and even sloughing of the fauces, will often occur in low states of the system from its use, and that yet these things frequently come on when not a particle has been given, as we have more than once had opportunities of witnessing.

Blisters, again, require a most cautious application: applied too early, they add to the fever and irritability of the system; too late, we have sloughing. Though we have seen several recoveries after this had happened.

Opiates appear to lock up the hepatic secretion, and often suppress the expectoration. We have often regretted their administration.

In treating of pneumonia and capillary bronchitis in this irregular sketch, we have grouped them together, because we wished to draw attention, more particularly to the complications which embarrass us in the treatment, and because the latter so frequently runs into the former, that we scarcely feel assured, from day to day, which disease the morrow may bring forth. Indeed, the two often merge into each other by an almost imperceptible line.

And we have referred to the difficulties attending us in the employment of the most prominent remedies, rather than endeavored to point out any particular course of treatment. Our aim has been, to be suggestive of obstacles, which being impressed upon the mind, will make us look clearly for the indications to be fulfilled, and weigh well the agents brought to bear upon the component parts of the disease.

A summary of the general plan of treatment which we have pursued, and we have done.

Vs. when the patient is seen early, to a decided extent; to be employed with extreme caution, when a repetition of it seems to be demanded, or when used late in the sickness; cupping, more particularly when a pleuritic stitch arises.

If the bowels are not loose, a full dose of calomel, taking care to insure a free evacuation of the intestinal canal by ol. ricini, if required.

Nauseant doses of ipecacuanha.

Opiates as seldom as possible. In many instances they are imperatively demanded by wakefulness, restlessness and pain, and to control the action of the bowels. We prefer full doses, when they are given, and that they should follow vs. at once, if the circumstances of the case will permit. When we can select the time of their administration, a little before the customary period for retiring to rest, is, for obvious reasons, the best.

Calomel, in doses decidedly alterative, and that will place us in a situation to produce ptyalism speedily, if we should, in the course of the disease, deem it expedient. If the disease remains mere bronchitis, we cannot conceive why the action of mercury should be pushed, nor if the abdominal complications should disappear from a pneumonic case, unless hepatization of the lung existed, would we consider it advisable.

Alkalies and demulcents have done us good service in all stages and forms.

As soon as the force of the arterial action is broken, blisters, *applied over the liver and stomach.*

Whenever a stimulant becomes admissible, quinine.

Later in the disease, stimulating expectorants, more especially the senega, combined with gradually increasing proportions of sanguinaria, still continuing the quinine. It has seemed preferable to us, that the quinine, in the advanced periods, should be given so as not to exceed from five to eight grains daily.

During many cases of convalescence from pneumonia, when all decided febrile action had passed, but consolidation remained, we have seen decided benefit from the internal use of the iodide of potassium, by no means a novel practice, but one concerning the efficacy of which many practitioners are sceptical.

We are, however, passing beyond the limits we had assigned ourselves, our intention having been, simply and briefly, to call attention to the manner in which inflammatory action of the lungs might be modified by preceding miasmatic exposure, and to enquire what modification of treatment might thereby become necessary.

*Western Branch, Norfolk Co.,*

*December*, 1852.

